# The ‘island effect’ in terrestrial global change experiments: a problem with no solution?

**DOI:** 10.1093/aobpla/plv092

**Published:** 2015-07-27

**Authors:** Sebastian Leuzinger, Simone Fatichi, Jarrod Cusens, Christian Körner, Pascal A. Niklaus

**Affiliations:** 1 Institute for Applied Ecology New Zealand, School of Applied Sciences, Auckland University of Technology, Private Bag 92006, Auckland 1142, New Zealand; 2 Institute of Environmental Engineering, ETH Zurich, Stefano Franscini Platz 5, 8093 Zurich, Switzerland; 3 Institute of Botany, University of Basel, Schönbeinstrasse 6, CH-4056 Basel, Switzerland; 4 Institute of Evolutionary Biology and Environmental Studies, University of Zurich, Winterthurerstrasse 190, 8057 Zurich, Switzerland

**Keywords:** DGVM, elevated CO_2_, FACE, hydrology, land–atmosphere coupling, vegetation feedback effects, warming

## Abstract

Most of the currently experienced global environmental changes (rising atmospheric CO_2_ concentrations, warming, altered amount and pattern of precipitation, and increased nutrient load) directly or indirectly affect ecosystem surface energy balance and plant transpiration. As a consequence, the relative humidity of the air surrounding the vegetation changes, thus creating a feedback loop whose net effect on transpiration and finally productivity is not trivial to quantify. Forcedly, in any global change experiment with the above drivers, we can only treat small plots, or ‘islands’, of vegetation. This means that the treated plots will likely experience the ambient humidity conditions influenced by the surrounding, non-treated vegetation. Experimental assessments of global change effects will thus systematically lack modifications originating from these potentially important feedback mechanisms, introducing a bias of unknown magnitude in all measurements of processes directly or indirectly depending on plant transpiration. We call this potential bias the ‘island effect’. Here, we discuss its implications in various global change experiments with plants. We also suggest ways to complement experiments using modelling approaches and observational studies. Ultimately, there is no obvious solution to deal with the island effect in field experiments and only models can provide an estimate of modification of responses by these feedbacks. However, we suggest that increasing the awareness of the island effect among both experimental researchers and modellers will greatly improve the interpretation of vegetation responses to global change.

## Abstract (precis)

The fact that we can only treat subsets of the population we are trying to make inferences on poses severe problems in any scientific discipline, primarily due to low replication. However, the sample's response may also be different from the population response due to what we call the ‘island effect’. Here, we systematically look at how global change experiments on vegetation may be subjected to this phenomenon, for example by treatment plots that are subjected to air whose characteristics (relative humidity, temperature) are influenced by the surrounding, non-treated vegetation. This may introduce a systematic artefact that experiments cannot account for.

## Introduction

**Figure 1.**
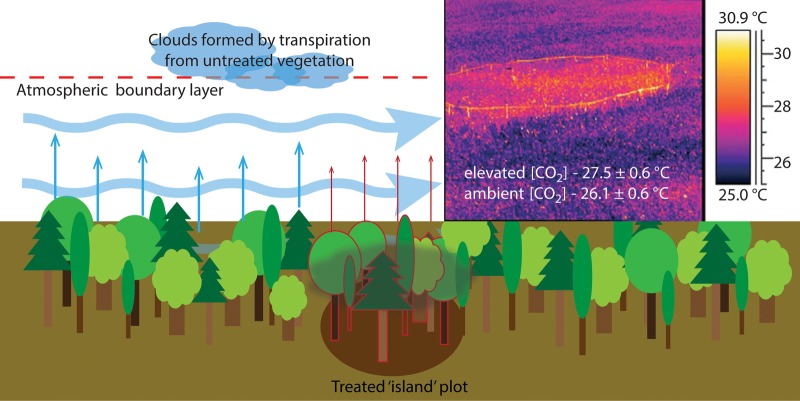
A simple representation of the ‘island effect’. The small plot of treated vegetation with, for example, lowered transpiration is surrounded by untreated vegetation with unaltered and relatively higher transpiration. Clouds formed by the untreated plots influence the treated vegetation via altered VPD and lowered solar radiation. The darkened soil below the treated plot illustrates the weak coupling of neighbouring plots relative to the strong coupling of atmospheric conditions. The insert on the top right clearly shows a change in surface energy balance due to lowered stomatal conductance in response to elevated CO_2_ (from Leakey 2009 reproduced with permission from The Royal Society).

A manipulative field experiment can treat only a small subset of the area or population in question. This inevitably poses the question ‘what would the results look like if we were able to subject the whole area, or the whole population to our treatment?’ The answer to this question will generally be ‘we don't know’. We term this caveat of being able to treat only small ‘islands’ the ‘island effect’ in its broadest sense (*sensu lato*). Examples probably exist even outside biological disciplines, but within ecology, they are omnipresent: if we treat a patch of grass with fertilizer, food quality may increase and attract herbivores. However, if the whole landscape was exposed to the fertilizer treatment, no particular attraction of herbivores to our small experimental plots would occur. Generally speaking, by treating small subsets of a larger entity we wish to study, we are missing certain processes or mechanisms. Here, we focus on a problem where the island effect is likely very significant, i.e. manipulative experiments that are used to simulate environmental conditions that vegetation is expected to experience in the future. Most commonly, atmospheric CO_2_ concentration, temperature, precipitation amount and patterns, and nutrient input are altered according to certain global change scenarios, and plant responses (e.g. transpiration or biomass accumulation) are recorded. Although the physiological mechanisms of the responses to some of these drivers are generally well understood at the smallest scale (e.g. the leaf-level response to varying CO_2_ concentrations has been successfully modelled since the early 1980s, see Farquhar *et al*. 1980; Jarvis *et al*. 1999), it is difficult to scale leaf-level or single tree responses to stand or regional levels. Still to date, physiological processes in response to global change drivers are often implicitly scaled from the leaf to the stand, catchment or region, ignoring the multitude of potential atmospheric, soil and community feedback processes (Körner 2000; Leuzinger and Hättenschwiler 2013). Generally, feedback processes that occur at a smaller scale than the treated plot can be captured experimentally, but not those occurring at scales exceeding the plot size. Thus, the interpretation of experiments that treat small ‘islands’ of vegetation (e.g. Shaw *et al*. 2002; Morgan *et al*. 2004; Norby *et al*. 2005; Kongstad *et al*. 2012; Bader *et al*. 2013) are necessarily based on the assumption that feedback processes acting beyond the plot size are not important. Belowground, this assumption is generally met because soil conditions induced by treatments are likely less influenced by the surrounding, untreated conditions. One exception may be changes in the ground water table, which we would only observe if a larger region was treated. However, unlike air, soil from ambient plots is not transported to the treatment plots (Fig. 1). An example for this is a change in fine root biomass in free-air CO_2_ enrichment (FACE) experiments (Iversen *et al*. 2008; Bader *et al*. 2009; Ellsworth *et al*. 2012): for instance, changed soil conditions due to increased fine root turnover in an elevated CO_2_ plot will directly affect the experimental plot, as no soil from the surrounding ambient vegetation is transported to the treatment plot. In contrast, the constantly moving air over experimental plots will likely be influenced by vegetation growing in ambient conditions outside the experimental plot (Fig. 1). For example, a cloud that was formed due to transpiration over one region will shade and cause stomatal closure in leaves of distant plants (Fig. 1) (van der Ent *et al*. 2010; Gimeno *et al*. 2012). Thus, if a global change driver (e.g. temperature or elevated CO_2_) leads to changes in transpiration in a given experimental plot, the feedback effects arising from this change cannot be ‘sensed’ by the vegetation in the plot.

While there may be countless ‘island effects’ (broad sense, as defined above), in the following, we focus on atmospheric water dynamics and call the imperative failure of any manipulative experiment to account for atmospheric feedback ‘island effect’ (*sensu stricto*, as used henceforth), referring to small ‘islands’ of treated plots in the ‘sea’ of atmospheric conditions dominated by ambient vegetation (Fig. 1). In short, an island effect is a plant or ecosystem response that is due to a feedback loop triggered by an initial change in stomatal conductance (*g*_s_), which we are missing in our experiments. In the long term, the island effect can also be triggered by changes in the surface energy balance, for example through changes in leaf area index (LAI) or plant community composition. The phenomenon may thus occur in any field experiment in which we manipulate factors that can affect *g*_s_ or the surface energy balance, such as experiments with elevated atmospheric CO_2_ (Ainsworth and Long 2005; Norby *et al*. 2010; Norby and Zak 2011), soil or air temperature changes (Melillo *et al*. 2002; Shaw *et al*. 2002), precipitation changes (Wu *et al*. 2011; Collins *et al*. 2012) and theoretically other factors such as nutrient availability (Krogman 1967) or ozone (Matyssek *et al*. 2010). The island effect may have regional and global implications for soil moisture, cloud formation, rainfall, run-off and finally plant productivity and vegetation structure, and any biogeochemical processes depending on these. Here, we first systematically characterize the island effect and then attempt to quantify its importance in various global change experiments. Finally, we provide an outlook on possible ways forward.

## Identifying the Problem

**Figure 2.**
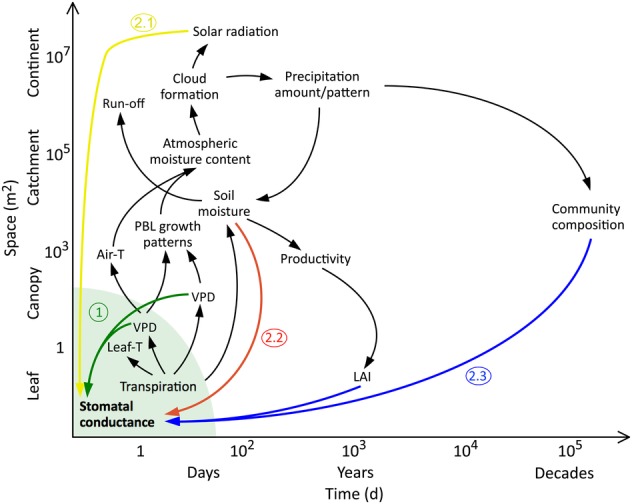
Potential feedback effects originating from an initial change in transpiration. In terrestrial global change experiments, these can turn into what we call ‘island effects’, depending on the considered spatial and temporal scale. At the lowest spatiotemporal scale (under the green arc), the island effect can be assumed to be zero, as the leaf boundary layer conditions are in fact influenced only by the respective leaf. The green arrows (1) represent first-order feedback effects, only leaf-level VPD is involved. Second-order feedback effects involve other factors such as cloud formation (2.1, yellow arrow), soil moisture (2.2, red arrow) or longer-term changes such as LAI or community composition (2.3, blue arrows).

Because the feedback loops associated with the island effect act across large spatial and temporal scales, the question of interest must be as follows: ‘what do we miss in an experiment due to the island effect?’ Admittedly, this question becomes almost infinitely complex and there is no definite answer, at least not from experiments. However, we can simplify the question by restricting ourselves to a given spatiotemporal scale (Fig. 2). All higher-order feedbacks missed in an experimentally treated vegetation island will have their origin in changes in transpiration caused by changes in *g*_s_. This is not quite true if long-term changes in LAI and community composition are considered, as those potentially entail changes in albedo, leaf energy balance and stand transpiration, irrespective of changes in *g*_s_. However, this long-term feedback can also arise from first-order changes in *g*_s_ (see group 2.3), under which category they will be treated here.

At otherwise constant microclimatic conditions, transpiration of a leaf simply depends on *g*_s_ and the vapour pressure deficit (VPD) in the boundary layer of that leaf (Jarvis and McNaughton 1986). If for now we ignore any transport of moisture away from the leaf, we can identify a positive feedback loop at the level of the leaf, initiated by a change in *g*_s_: for example, if water addition to the soil increases *g*_s_, transpiration will increase and the boundary layer VPD will decrease, which in turn increases stomatal conductance, decreases leaf temperature and so forth (loop under green area in Fig. 2). Thus, at the smallest spatiotemporal scale, there is no island effect, since all relevant feedbacks occur within the plot's size and the experiment's duration. Put simply, under these assumptions, the air ‘created’ or ‘influenced’ by the leaf is the air that the leaf ‘sees’ or is exposed to. However, as soon as we extend our spatial and/or temporal perspective, this is no longer true (area outside the green area in Fig. 2). For example, at landscape scale, the air that a leaf ‘sees’ may well have been affected (e.g. in terms of its moisture content or temperature) by plant transpiration hundreds of kilometres away. The plethora of potential feedbacks at these larger scales can roughly be grouped them into (1) first-order and (2) second-order or higher-order effects. Group (1) includes effects where *g*_s_ affects transpiration and thus VPD, which then immediately feed back to *g*_s_—no further variables are involved (green arrows in Fig. 2). Group (2) includes effects that are more complex as they involve at least one more secondary effects before they feed back to *g*_s_: (2.1) those caused by changes in solar radiation, via alteration of atmospheric moisture and therefore cloud cover (de Arellano *et al*. 2012) (yellow arrow in Fig. 2); (2.2) those caused by changes in soil moisture, via first-order feedbacks through VPD and transpiration (red arrow in Fig. 2) and (2.3) effects that follow a few to many years after an initial change in *g*_s_, either via changes in LAI, adaptive responses of leaf and canopy conductance or eventually soil biogeochemistry and community composition (blue arrows in Fig. 2). Note again that all loops in Fig. 2 originate from, and feed back to, changes in stomatal conductance per unit leaf area as the ultimate hub of all island effects. Those feedback loops that operate outside the influence of *g*_s_ may be important, but are ‘dead ends’, because they are not prone to further altering the island effect.

**Figure 3.**
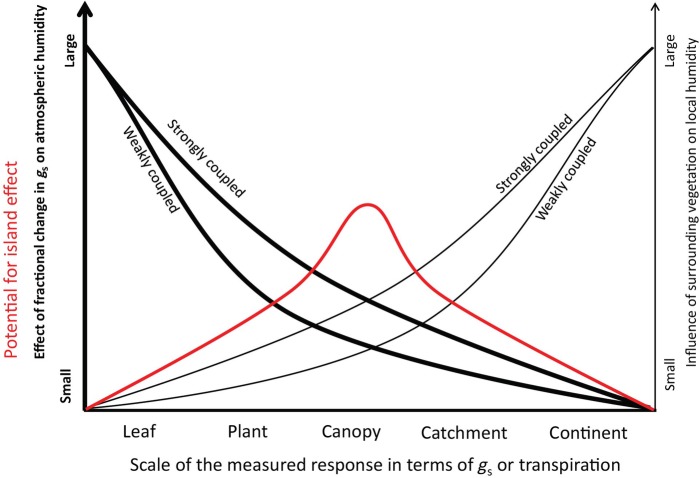
The potential for the island effect is maximal at intermediate spatial scales. This is because the two effects shown (effect of *g*_s_ on atmospheric humidity, influence of surrounding vegetation on local humidity) are compensatory in terms of promoting the island effect. Generally, atmospherically well-coupled plants/stands such as tree canopies are more prone to the island effect than less coupled ones (e.g. grassland, see McNaughton and Jarvis 1991).

Can we identify at what spatiotemporal scale we are most likely to see an island effect in an experiment? The VPD conditions a leaf or plant ‘sees’ are a blend of its own transpiration as well as that of the neighbouring leaves, plants, stand, region or continent. On the one hand, the larger the spatial scale at which potential feedback effects act, the higher the risk that we miss them in a plot-size experiment. On the other hand, at increasing scales (from <1 m^3^ to many km^3^), the influence of a fractional change in *g*_s_ on large-scale transpiration decreases (Fig. 3). This is because the bulk of Earth's evapotranspiration originates from oceans, with plant transpiration accounting for only ∼9–10 % of total water vapour input to the atmosphere (Roderick *et al*. 2014; Schlesinger and Jasechko 2014; Wild *et al*. 2015). Additionally, the feedbacks (and thus the risk of an island effect) are more pronounced in vegetation that is strongly coupled to the atmosphere, corresponding to a small aerodynamic resistance (Fig. 3) (Jarvis and McNaughton 1986).

In summary, the two needed ‘ingredients’ or prerequisites for the island effect to occur are (i) a first-order change in *g*_s_ that causes a change in transpiration following a global change treatment and (ii) that the air surrounding the leaf/plant/stand of interest is influenced to some degree by surrounding, non-treated leaves/plants/stands. In the following, we work through the most important experiments with global change drivers, characterizing where and when island effects may occur. For the rest of the text, we refer to the above-defined categories, which are also used in Fig. 2 (labelled 1, 2.1, 2.2 and 2.3).

## Which Experiments Are Affected?

As long as the two ingredients for the island effect are present, it does not matter what triggers the first-order change in transpiration. The most prominent drivers, however, are elevated CO_2_, warming, precipitation and nutrient addition/depletion experiments. Once an initial change in *g*_s_ has occurred, we need not worry much about what lies at the origin of this change, as the feedback effects (or the island effect as seen from the experimenter's perspective) will be identical. We will now briefly review what initial changes in *g*_s_ have been found following experiments with the most common global change treatments.

Free-air CO_2_ enrichment experiments have been employed for >20 years testing responses of ecosystems to elevated atmospheric CO_2_ (Ainsworth and Long 2005). Generally, reduced stomatal opening is observed when plants are subjected to elevated atmospheric CO_2_, leading to lower levels of transpiration, i.e. decreased latent heat flux. Most meta-analyses report responses of stomatal conductance to elevated CO_2_ between −30 % and no response, with a mean around −15 % (at approximately doubled pre-industrial atmospheric CO_2_ concentration), strongly depending on the species and testing conditions (Field *et al*. 1995; Curtis and Wang 1998; Medlyn *et al*. 2001; Ainsworth *et al*. 2003; Ainsworth and Rogers 2007; Leuzinger and Körner 2007; Keel *et al*. 2007; Warren *et al*. 2011; Tor-ngern *et al*. 2015). In a nutrient-poor calcareous grassland, Niklaus and Körner (2004) found a ∼50 % reduction in leaf conductance in the dominant grass species, and no response in co-occurring sedges sharing the same root sphere. These strong responses in *g*_s_ are, therefore, likely to trigger experimental island effects.

Warming experiments use several methods to heat the soil, usually with buried heating cables (e.g. Rustad *et al*. 2001; Melillo *et al*. 2002), or the atmosphere, usually with infrared lamps (e.g. Nijs *et al*. 1996; Hovenden *et al*. 2006; De Boeck *et al*. 2010). Plants respond to soil warming differently, depending on the biome with some reporting elevated *g*_s_ (Rogiers and Clarke 2013) and others a decrease in *g*_s_. The net effect of warming on surface energy balance seems to be important: Nijs *et al*. (1997) found, for example, that despite a reduction in *g*_s_, leaf transpiration was elevated because the warmed canopy increased leaf-to-air vapour pressure difference. Trees in boreal forests may have stimulated *g*_s_, particularly in the morning and early spring, whereas tropical trees may not be affected much by temperature in terms of *g*_s_ (Doughty and Goulden 2008). In cooler areas like the temperate zone, elevated soil temperatures have been shown to increase *g*_s_ (e.g. Rogiers and Clarke 2013). Under well-watered conditions, warming can increase night-time *g*_s_ and/or sap flow because of increased VPD, which can contribute considerably to total transpiration (Caird *et al*. 2007; Fisher *et al*. 2007). Furthermore, atmospheric warming increases VPD, which, everything else being equal, increases transpiration. This may evoke a potential feedback effect that is not immediately linked to *g*_s_. However, to avoid excessive water loss and/or cavitation, plants often lower *g*_s_ in response to high transpiration or low leaf water potentials (e.g. Bunce 1997; Tardieu and Simonneau 1998; Brodribb and Holbrook 2003). Also, immediate canopy warming may be a feedback loop acting at small scales (under the arc in Fig. 2) and thus not evoke an island effect. Warming can also affect phenology (Keenan *et al*. 2014), with consequences for seasonal transpiration rates. For example, Zavaleta *et al*. (2003) found that under warming, soil water content in annual grassland increased not due to reduced transpiration at the leaf level, but a reduction in seasonal transpiration via an early onset of senescence. In this case, a potential island effect would act via transpiration, as phenology changes alone cannot cause an island effect.

Precipitation and drought experiments eventually alter soil water availability to plants. The influence of water availability on *g*_s_ is well established (Hsiao 1973; Schulze *et al*. 2002; Dodd 2013); declining soil moisture reduces *g*_s_, whereas increasing soil moisture may elevate *g*_s_ when plants are water limited. In addition, stomatal responses vary strongly between species so that elevated CO_2_ can trigger shifts in community composition via water use-related mechanisms (Volk *et al*. 2000; Morgan *et al*. 2011). Therefore, predicting indirect ecosystem responses to elevated CO_2_ requires a knowledge of the species’ characteristic stomatal regulation.

Finally, nutrient addition experiments have a long history in ecology, with growth and photosynthesis as typically measured responses. Increased productivity mostly translates to higher LAI, and possibly canopy conductance (at least at LAI <3, Krogman 1967; Schulze *et al*. 1994; Novick *et al*. 2009). Along with LAI come changes in albedo and surface energy balance. These changes likely occur over longer time periods, and the resulting feedbacks will be of group 2.3 in Fig. 2. Only if ultimately *g*_s_ or transpiration are affected, which is likely the case, can an island effect (*sensu strico*) occur as a consequence of nutrient addition.

## What Are We Missing?

It is clear from our above discussion that many global change drivers will, among other, cause changes in *g*_s_ and/or transpiration, inevitably leading to changes in the atmospheric moisture conditions immediately surrounding the plant. If the scale of the feedback mechanism exceeds the plot scale, these new atmospheric conditions do not feed back on the plants in the treated plot, producing an island (Fig. 2). If, hypothetically, we could treat a large enough area so that the entire vegetation cover affected by the simulated global change driver transpired differently as in a realistic scenario, plants could in fact be subjected to a series of both positive and negative feedback mechanisms, with an unknown net outcome (Fig. 2).

A first, immediate and positive feedback loop could arise when, for example, drier air causes higher evaporative demand, leading to further stomatal closure and thus enhancing the first-order response (effect 1 in Fig. 2). This mechanism was indeed found early in a modelling attempt by Jacobs and de Bruin (1997), who used a planetary boundary layer (PBL)–vegetation model to simulate the vegetation–atmosphere interaction under elevated CO_2_. The problem is that an opposite effect is just as plausible, as higher VPD typically causes more transpiration despite stomatal closure. More generally, even if we are able to isolate one possible feedback as these authors did, other, less immediate effects acting at different spatiotemporal scales may overlay and thus cancel, mitigate or enhance the initial one. For example, on a spatial scale of a few to tens of kilometres, larger sensible heat flux caused by reduced transpiration could cause a decrease or even increase in cloud formation (D'Almeida *et al*. 2007; Knox *et al*. 2011), leading to modified radiation and thus altered surface energy balance, altered evapotranspiration and also altered stomatal conductance (effect 2.1 in Fig. 2). Other negative feedback loops could arise via reduced rain (following decreased cloud formation) or higher evaporative demand, both leading to lower soil moisture and eventually lower *g*_s_ (effect 2.2 in Fig. 2). It is important to note that the latter two phenomena (effects on soil moisture via atmospheric feedback loops) are somewhat different from the direct influence of reduced *g*_s_ on soil moisture, an effect that can actually be captured in an experiment (see below). The atmospheric island effect may, therefore, lead to an over- or underestimation, or even a sign reversal of the first-order response in terms of *g*_s_ reported from an experiment.

While the island effect is inevitable aboveground, we can look at what happens belowground, where there is no island effect (*sensu stricto*). Soil, unlike air, is not transported to roots from far away, untreated vegetation in global change experiments. Apart from edge effects, the soil water conditions (e.g. soil moisture) that the roots ‘create’ are directly acting on them. If a tree transpires less due to elevated CO_2_ for example, it will cause the soil to be moist for longer. The magnitude of this measurable feedback effect in the soil, however, can be very large, to the point that increased soil moisture following a reduction in *g*_s_ in FACE experiments often causes larger growth stimulation than the direct effects of elevated CO_2_ (Housman *et al*. 2006; Holtum and Winter 2010; Hartmann 2011). This is particularly true in grassland. In semi-natural grassland, lower stomatal conductance leads to significant soil moisture savings (Niklaus *et al*. 1998), which explained almost the entire variation in peak biomass CO_2_ response (Niklaus and Körner 2004). Similar effects were also found in other studies (Owensby *et al*. 1997; Morgan *et al*. 2004; Hovenden *et al*. 2014). In other words, if an island effect existed belowground, i.e. if the soil moisture that the roots of our CO_2_-treated plants experience came from ambient vegetation, we would be far off with our conclusions of ecosystem responses to elevated CO_2_! Are we missing something similar aboveground where we actually have to deal with the island effect? Probably not, but even if the island effect is much smaller than the elevated CO_2_–soil feedback effect we can measure, we would be likely to observe different net ecosystem responses.

Ultimately, the issue of the island effect will involve large temporal scales, as is often the case in ecology (Leuzinger *et al*. 2011). Effects acting over long time spans have the power to change the community structure or soil biota and therefore the nutrient cycles. These are very difficult to model or even to speculate about.

## Ways Forward

We argue that the island effect is primarily a phenomenon that we need to be aware of when interpreting both experimental and modelling results. To date, few researchers are. No individual experiment or model will provide a conclusive answer, but by intelligently combining experimental and modelling approaches, we can better understand their sensitivities to the island effect. At the global scale, a fully coupled model technically takes care of the island effect, as vegetation is sensitive to changes in temperature, CO_2_, soil moisture, radiation, etc. and dynamically feeds back to the atmosphere (Cao *et al*. 2010). However, the coarse resolution and representation of vegetation diversity of such global models means that most of the above feedbacks will be only partially represented, and we will have to rely on more detailed vegetation modelling (e.g. Pappas *et al*. 2015*a*, *b*).

Because the island effect almost always means missing feedback on *g*_s_ and therefore transpiration and surface energy balance, one crucial point of attack will be to better characterize VPD sensitivities. We know water fluxes may be ‘wrong’ (which cannot be fixed due to limitations in plot size), but we need to know whether the parameter investigated is sensitive to the changes in water fluxes that occur. This could be achieved by implementing factorial treatments that modify water availability. For example, a factorial CO_2_ × irrigation study could indicate that CO_2_ effects are dependent on water availability. If, for example, CO_2_ effects disappear when the soil moisture saving that occurs in experimental plots is offset (e.g. by equivalent water addition to control plots), then we know that we potentially have a problem. If the CO_2_ effects on a given variable remain largely constant, then the inferences are probably safe and not affected by the island effect. Another way to understand VPD responses are experiments where relative humidity (RH) is manipulated. Misting has been used in agriculture and horticulture (e.g. Katsoulas *et al*. 2001) to reduce leaf temperatures and transpiration, but RH experiments are rare in global change research and we know of just one in Tartu, Estonia (Kupper *et al*. 2011; Godbold *et al*. 2014). Kupper *et al*. (2011) found that higher atmospheric humidity increased both sap flow and canopy conductance, while Godbold *et al*. (2014) found that leaf longevity increased in one of two species measured in elevated RH. Taken together, these results suggest that annual transpiration can be higher in elevated RH conditions in this experiment, despite lower VPD. Another experimental approach to at least estimate the magnitude of a possible island effect could be to water different size (grassland) plots, e.g. from 1 m^2^ to several km^2^. Although watering even very large areas will not affect the diurnal PBL growth, if the plot size is the only variable that changes between plots of different sizes, we should see differences in ecosystem responses that are simply due to the island effect.

**Figure 4.**
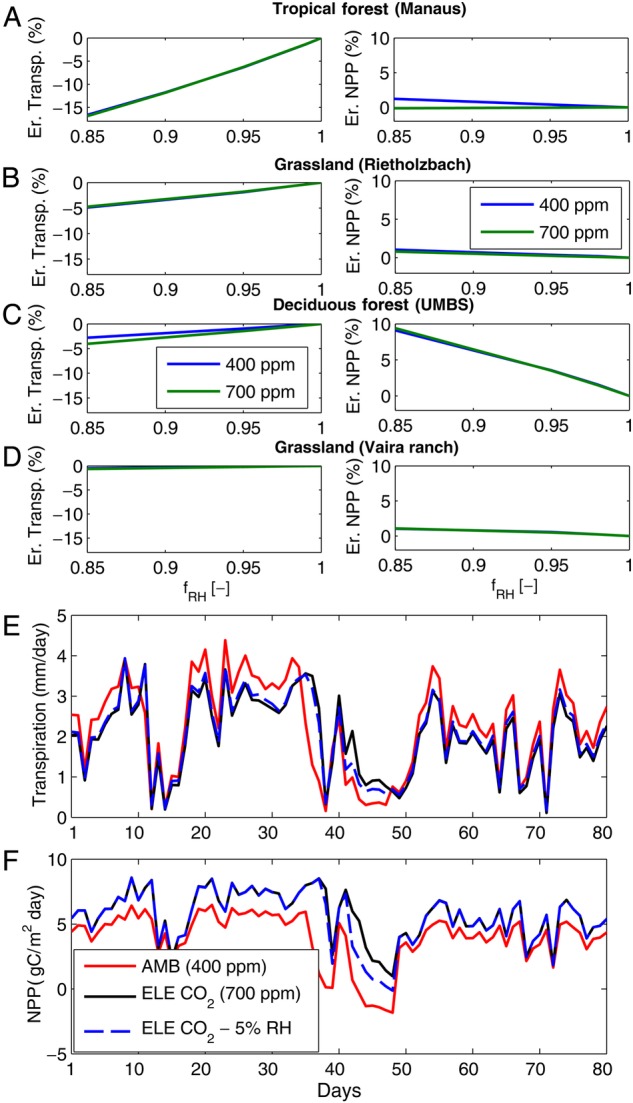
Sensitivity of the island effect in an elevated CO_2_ experiment. The potential error (Er.) committed in estimating transpiration and NPP with the current RH is shown. RH is reduced by a given factor (*f*_RH_), due to hypothetical local or regional feedbacks. The numerical experiment is carried out for two CO_2_ levels: 400 ppm (ambient, AMB) and 700 ppm (elevated, ELE CO_2_) and four locations: a tropical forest in Manaus (Brazil), a grassland in Rietholzbach (Switzerland), a deciduous forest near the University of Michigan Biological Station (UMBS) Michigan, USA, and a grassland in California, USA (Vaira ranch). The simulated time series of transpiration and NPP during a characteristic growing season at the UMBS are also shown (subplots E and F); note the dry period between days 2040 and 2050.

To constrain the potential bias that the island effect introduces in experiments, explicit simulations of changes in VPD using coupled vegetation–atmosphere models could help. As an example, we used a mechanistic ecohydrological model, T&C (Fatichi *et al*. 2012, 2015; Fatichi and Ivanov 2014), to carry out a sensitivity analysis of the potential errors one can commit assuming that RH does not change in a manipulation experiment, where, instead, a reduction in *g*_s_ is expected (e.g. elevated CO_2_). We applied two CO_2_ levels (400 and 700 ppm), and different reduction factors (*f*_RH_) to the observed time series of RH, from *f*_RH_ = 1.0 (no reduction) to *f*_RH_ = 0.85 corresponding to a strong (−15 %) reduction of RH. We used four locations characterized by different climates and plant functional types to show the potential variability of the effect (Fig. 4A–D). Simulated errors in the long-term (5–10 years) transpiration may reach −5 to −10 %, but they are generally constrained to less than −2 % for realistic changes in RH (Fig. 4). The negative sign is expected since the island effect leads to an underestimation of transpiration because RH is not reduced. Simulated errors in net primary productivity (NPP) are constrained within ±2 %, except for one case study (UMBS in Fig. 4C), where they can reach up to +10 %. For this location, the changes in transpiration due to a lower RH are sufficient to increase the water stress in the forest stand with comparison to unchanged RH. Changes in soil moisture considerably affect NPP, which would be overestimated in the treated ‘island’. While the long-term expected errors may be small, there are specific seasons or periods where the island effect can be potentially very significant (>100 %), for example the dry period in the middle of the growing season (days 40 to 50, Fig. 4E and F). In this scenario, the difference in transpiration and NPP induced by a 5 % change in RH can affect the system response of a magnitude similar to that of an elevated CO_2_ treatment. Since many observations in manipulation experiments are typically carried out for limited periods during the growing season, the potential artefacts of the island effect may be significant. The presented results are a sensitivity analysis and likely dependent on model parameters, but they suggest that there may be situations or locations, where a small change in RH can lead to considerably different plant water stress, with potentially large implications in terms of ecosystem stability and composition.

## Conclusions

In conclusion, we advocate an increasing awareness for the systematic biases that may emerge from plot-size dependence of responses to simulated global change. Generally, it appears imperative that future global change field experiments are designed in a way that enables effective up-scaling strategies or that at least allow to constrain the magnitude of potential island artefacts. Ideally, these bias estimates are then published together with the study results. Modelling efforts to explicitly simulate the magnitude of the island effect dependent on the affected plot size could help to successfully tackle this challenging endeavour. Water-vapour-related scaling issues have now been discussed for almost three decades (Jarvis and McNaughton 1986; Amthor 1999), but in our opinion, the problem has not been effectively dealt with to date. We believe that this situation could be overcome by adopting approaches along the lines we discussed here and by explicitly dealing with the island effect when designing experimental studies or when using field experimental data to parameterize models used for regional or global simulations. While models are often based on physical laws (e.g. energy conservation) and physiological principles (e.g. the photosynthesis scheme based on the Farquhar model), special attention needs to be given to the degree to which they might have been implicitly or explicitly ‘tuned’ to match experimental data (Leuzinger and Thomas 2011)—experimental data which, as we stress here, might suffer from bias due to the island effect. Such a ‘trickling’ of bias into models is likely since models are abstractions that necessarily capture only part of the real-world complexity, and are gauged against experimental data. Most ecosystem models are based on a ‘carbon-centred’ approach and only implement a limited number of feedback mechanisms that may dampen this ‘C-driven’ (i.e. photosynthetic) response (Leuzinger *et al*. 2011; Fatichi *et al*. 2014). This general issue has been recognized (Hungate *et al*. 2003; Piao *et al*. 2013), but our understanding is that the island effect in experimental data against which models are gauged has been largely ignored so far.

## Sources of Funding

J.C. acknowledges the supported provided by a Vice Chancellor's Doctoral Scholarship. S.L. and S.F. received a travel grant from the institute of Applied Ecology New Zealand, Auckland University of Technology. P.A.N. is supported by the University of Zurich Priority Programme on Global Change and Biodiversity.

## Contributions by the Authors

S.L., P.A.N. and C.K. conceived the idea. S.F. performed the model analyses. J.C. developed the idea further and drafted the section ‘Which Experiments Are Affected’. All authors wrote the manuscript.

## Conflict of Interest Statement

None declared.
